# Redefining Infarction Size for Small-Vessel Occlusion in Acute Ischemic Stroke: A Retrospective Case–Control Study

**DOI:** 10.3390/neurolint16050088

**Published:** 2024-10-21

**Authors:** Yen-Chu Huang, Hsu-Huei Weng, Leng-Chieh Lin, Jiann-Der Lee, Jen-Tsung Yang, Yuan-Hsiung Tsai, Chao-Hui Chen

**Affiliations:** 1 Department of Neurology, Chang Gung Memorial Hospital at Chiayi, Chiayi 613, Taiwan; jdlee540908@cgmh.org.tw (J.-D.L.); trianglescorpio@gmail.com (C.-H.C.); 2 College of Medicine, Chang-Gung University, Taoyuan 333, Taiwan; jents716@ms32.hinet.net (J.-T.Y.); russell.tsai@gmail.com (Y.-H.T.); 3 Department of Diagnostic Radiology, Chang Gung Memorial Hospital at Chiayi, Chiayi 613, Taiwan; hweng@post.harvard.edu; 4 Department of Emergency Medicine, Chang Gung Memorial Hospital at Chiayi, Chiayi 613, Taiwan; a3456711@ms65.hinet.net; 5 Department of Neurosurgery, Chang Gung Memorial Hospital at Chiayi, Chiayi 613, Taiwan

**Keywords:** small subcortical infarction, small-vessel disease, MRI, early neurological deterioration, small-vessel occlusion

## Abstract

Background/Objectives: Small-vessel occlusion, previously referred to as lacunar infarcts, accounts for approximately one-third of all ischemic strokes, using an axial diameter of less than 20 mm on diffusion-weighted imaging. However, this threshold may not adequately differentiate small-vessel occlusion from other pathologies, such as branch atheromatous disease (BAD) and embolism. This study aimed to assess the clinical significance and pathological implications of acute small subcortical infarctions (SSIs) based on infarct diameter. Methods: We conducted a retrospective case–control study using data from stroke patients recorded between 2016 and 2021 of the Stroke Registry in Chang Gung Healthcare System. Patients with acute SSIs in penetrating artery territories were included. Key variables such as patient demographics, stroke severity, and medical history were collected. Infarcts were categorized based on size, and the presence of early neurological deterioration (END) and favorable functional outcomes were assessed. Results: Among the 855 patients with acute SSIs, the median age was 70 years and the median National Institutes of Health Stroke Scale (NIHSS) score at arrival was four. END occurred in 97 patients (11.3%). Those who experienced END were significantly less likely to achieve a favorable functional outcome compared to those who did not (18.6% vs. 59.9%, *p* < 0.001). The incidence of END increased progressively with infarct sizes of 15 mm or larger, with the optimal threshold for predicting END identified as 15.5 mm and for BAD, it was 12.1 mm. A multiple logistic regression analysis revealed that motor tract involvement [adjusted odds ratio (aOR) 2.3; 95% confidence interval (CI) 1.1–4.7], an initial heart rate greater than 90 beats per minute (aOR 2.3; 95% CI 1.2–4.3), and a larger infarct size (15 mm to less than 20 mm vs. 10 mm to less than 15 mm; aOR 3.0; 95% CI 1.4–6.3) were significantly associated with END. Conclusions: Our findings suggest that setting the upper limit for small-vessel occlusion at 15 mm would be more effective in distinguishing it from BAD. However, these findings should be interpreted in the context of the retrospective design and study population. Further multi-center research utilizing high-resolution vessel wall imaging is necessary to refine this threshold and enhance diagnostic accuracy.

## 1. Introduction

Small-vessel occlusion, formerly referred to as lacunar infarcts, comprises approximately one-third of all ischemic strokes [1]. However, lacunar infarcts are not solitary cerebrovascular events; they often constitute a part of the spectrum of manifestations seen in cerebral small-vessel disease. This condition can be initiated by various pathologies, primarily hypertensive arteriopathy and cerebral amyloid angiopathy, both associated with endothelial dysfunction, blood–brain barrier degradation, inflammation, and other contributing factors [2,3]. Moreover, acute small subcortical infarctions (SSIs) can also arise from other causes, including branch atheromatous disease (BAD), intracranial artery stenosis, and embolism [3,4].

In the Trial of Org 10,172 in Acute Stroke Treatment (TOAST) classification, acute SSIs were categorized as small-vessel occlusions when the axial diameter was less than 15 mm [5]. This upper limit was revised to 20 mm in diffusion-weighted imaging (DWI) for small-artery occlusion in the Stop Stroke Study (SSS)–TOAST classification [6] or for a recent SSI in cerebral small-vessel disease [7]. However, specific infarct patterns such as a tubular shape, larger infarct size, or proximal location may suggest a BAD pathology [8,9,10]. While certain SSIs with BAD pathology may fit the current definition of small-artery occlusion, from a pathological standpoint, BAD should be classified as intracranial atherosclerosis and may warrant more aggressive treatment to reduce END and improve clinical outcomes [11,12]. This inconsistency could potentially complicate stroke classification and impact clinical research and treatment strategies.

In the Chinese ischemic stroke subclassification, BAD is distinctly categorized as large-artery atherosclerosis, while small-artery occlusion, now renamed as penetrating artery disease in this classification, is defined as an acute isolated infarct in a clinically relevant territory of one penetrating artery, regardless of the size of the infarct and without evidence of atherosclerotic plaque detected by high-resolution MRI [13].

However, visualizing these microatheromas or junctional plaques requires high-resolution vessel wall imaging, which is a time-consuming and impractical procedure for routine clinical application [14]. Consequently, BAD is often characterized by a large infarct size (>15 mm), proximity to the parent artery, or a tubular shape [4]. Nevertheless, the current definition, which relies on an arbitrary cut-off of 20 mm for small-vessel occlusion, is not entirely satisfactory, as it encompasses a significant number of cases involving BAD or embolic infarction.

The objective of this study is to assess the clinical relevance and possible pathologies of acute SSIs based on their infarct diameter.

## 2. Materials and Methods

**Figure 1:**
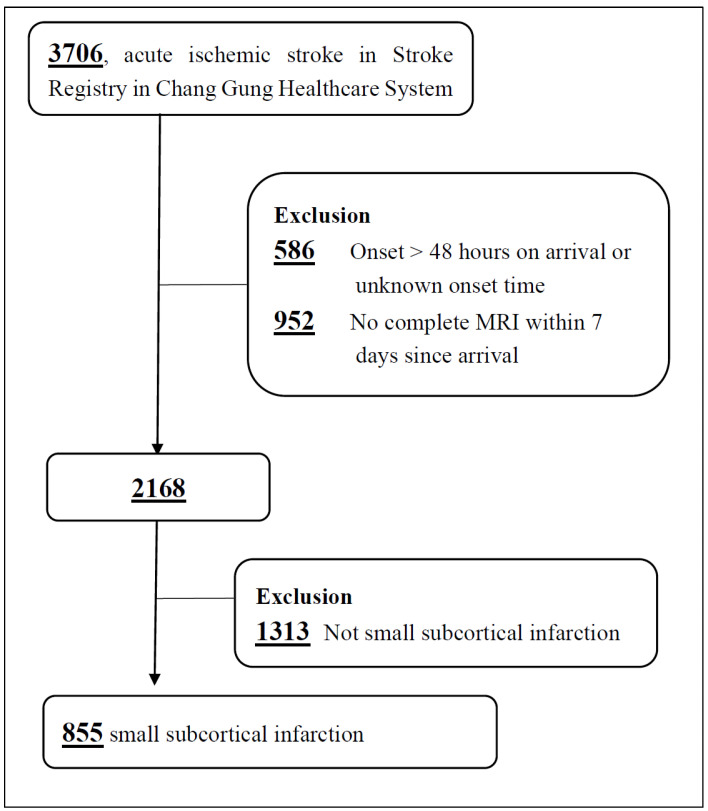
Study flowchart of patient selection.

This retrospective case–control study utilized data from stroke patients recorded between 2016 and 2021 at the Chiayi branch of the Stroke Registry in Chang Gung Healthcare System (SRICHS), Chiayi, Taiwan [15]. Clinical information for these acute ischemic stroke patients was gathered prospectively. Patients were excluded if their stroke onset time was unknown, they arrived more than 48 h post-onset, or did not undergo MRI within seven days of onset (Figure 1). The analysis focused on those with SSIs in penetrating artery territories with a maximum lesion size of 40 mm.

Key variables, including patient demographics, stroke severity, and medical history, were collected upon admission. The medical history encompassed conditions such as diabetes mellitus, hypertension, coronary artery disease, atrial fibrillation, congestive heart failure, hyperlipidemia, and prior strokes. Comprehensive MRI evaluations were performed on all patients, including extracranial and intracranial angiography via time-of-flight (TOF) techniques, and carotid and transcranial duplex scans were used to detect large-vessel pathologies. The decision to perform extended EKG monitoring or echocardiography was made by the attending physician. In addition, certain patients underwent investigations for occult atrial fibrillation using serial 12-lead electrocardiography, 24 h Holter monitoring, or echocardiography. For patients under 45, further assessments were carried out to determine potential causes of stroke at a young age. Stroke subtypes were classified based on TOAST criteria, which include large-artery atherosclerosis, cardioembolism, small-vessel occlusion, undetermined etiology, and other specific causes [5]. The small-vessel occlusion subtype was defined by an SSI within penetrating artery territories, with a maximum diameter of 20 mm on DWI. Early neurological deterioration (END) was defined as an increase of 4 or more points on the National Institutes of Health Stroke Scale (NIHSS) within 72 h of stroke onset [16]. A modified Rankin Scale score of ≤1 at 3 months was considered a favorable outcome.

BAD was diagnosed based on two criteria, namely (1) infarctions visible on four or more axial MRI slices (slice thickness 5–7 mm) within the lenticulostriate artery territory or extending from the basal surface of the pons within the paramedian pontine artery territory and (2) the absence of significant large-artery stenosis (>50%) or occlusion and no evidence of cardiogenic embolism [4,17]. Corticospinal tract involvement has been linked to the prediction of END. Therefore, we defined motor tract involvement as the presence of an acute infarct in specific regions, namely the posterior part of the corona radiata adjacent to the lateral ventricle, the posterior limb of the internal capsule, or the basal surface of the pons [18,19]. The imaging markers of cerebral small-vessel disease, such as cerebral microbleeds, enlarged perivascular spaces (EPVSs), and white matter hyperintensities (WMH), were also evaluated [20].

MRI data were obtained using either a 1.5-Tesla Philips Gyroscan Intera scanner (Philips Medical Systems, Best, The Netherlands) or a 3-Tesla Siemens Verio MRI system (Siemens Medical System, Erlangen, Germany), including standard sequences such as axial DWI, the apparent diffusion coefficient, T1- and T2-weighted images, fluid-attenuated inversion recovery images, and 3D TOF angiography covering the extracranial carotid artery and the circle of Willis.

### Statistical Analyses

Statistical analyses were conducted using SPSS software (version 27, Chicago, IL, USA). For continuous variables, the Kruskal–Wallis test was applied, while Fisher’s exact test or Pearson’s chi-square test was used for categorical data. When significant differences were identified across the five infarct sizes, post hoc comparisons between groups were performed using Mann–Whitney or chi-square tests with Bonferroni correction. Receiver-operating characteristic (ROC) curve analysis was utilized to determine the optimal infarct diameter cut-off for predicting END, BAD, and large-artery atherosclerosis. To adjust for baseline confounders, multivariate logistic regression analysis was performed for END and favorable outcomes. A two-tailed *p*-value of less than 0.05 was considered statistically significant.

## 3. Results

A total of 3706 stroke patients were identified from the Stroke Registry in Chang Gung Healthcare System (SRICHS) at the Jiayi branch between 2016 and 2021 (Figure 1). Out of these, 586 patients whose stroke onset occurred more than 48 h before arrival, as well as 952 patients who did not undergo a complete MRI study, were excluded from the analysis. This left 2168 patients, of whom 855 had acute SSIs.

For the 855 patients with acute SSIs, the median age was 70 years (61–78) and the median NIHSS score at the time of arrival was four (2–6). Among these patients, 329 (38.5%) had brainstem strokes, 97 (11.3%) experienced END, and 36 (4.2%) received intravenous alteplase treatment. At the three-month follow-up, 465 patients (54.4%) achieved a favorable functional outcome. Patients who experienced END were significantly less likely to achieve a favorable functional outcome compared to those who did not experience END (18.6% vs. 59.9%, *p* < 0.001). According to the TOAST classification, most patients were identified as having small-vessel occlusion (*n* = 582, 68.1%), followed by large-artery atherosclerosis (*n* = 104, 12.2%), cardioembolism (*n* = 72, 8.4%), undetermined causes (*n* = 94, 11.0%), and other causes (*n* = 3, 0.4%).

Table 1 outlines the baseline clinical characteristics based on infarct size. Patients with infarcts smaller than 10 mm had the mildest initial NIHSS scores at arrival and on the third day, the lowest incidence of BAD, the lowest incidence of END, and the highest rate of favorable outcomes at three months. Stroke risk factors, including hypertension, diabetes mellitus, atrial fibrillation, congestive heart failure, or previous stroke, did not show significant differences across various infarct sizes. Similarly, the incidence of cardioembolism was consistent across different infarct sizes. However, patients with infarct sizes of 25 mm or larger had the highest rates of large-artery atherosclerosis, END, and intravenous alteplase treatment. For small-vessel disease imaging markers, patients with infarcts smaller than 10 mm had the lowest WMH scores in both the basal ganglia and periventricular areas, as well as the lowest EPVS scores in the basal ganglia. Specifically, patients with infarcts smaller than 10 mm had significantly lower WMH scores in the periventricular area compared to those with infarcts measuring 20 mm to less than 25 mm (1.8 vs. 2.3, adjusted *p* = 0.044). Similarly, WMH scores in the basal ganglia (1.6 vs. 2.2, adjusted *p* < 0.001) and EPVS scores in the basal ganglia (1.6 vs. 2.2, adjusted *p* = 0.001) were also lower in patients with infarcts smaller than 10 mm compared to those with infarcts measuring 20 mm to less than 25 mm.

**Figure 2:**
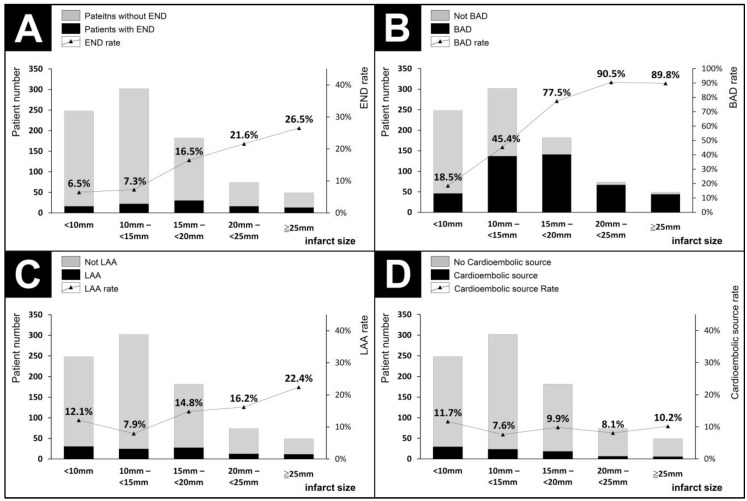
Number and percentage of patients with early neurological deterioration, branch atheromatous disease, large-artery atherosclerosis, and cardioembolism across different infarct sizes. (**A**) Early neurological deterioration (END). (**B**) Branch atheromatous disease (BAD). (**C**) Large-artery atherosclerosis (LAA). (**D**) Cardioembolism.

Figure 2 presents the percentage of patients with END, BAD, large-artery atherosclerosis, and cardioembolism across different infarct sizes. The incidence of END increased progressively with infarct sizes of 15 mm or larger. Patients with infarct sizes between 15 mm and less than 20 mm had a higher rate of END compared to those with infarct sizes between 10 mm and less than 15 mm (16.5% vs. 7.3%, adjusted *p* = 0.02). The optimal threshold for predicting END was found to be 15.5 mm. The incidence of BAD also rose with increasing infarct size, with significantly higher rates in the following comparisons: less than 10 mm vs. 10 mm to less than 15 mm (15.8% vs. 45.4%, adjusted *p* < 0.001) and 10 mm to less than 15 mm vs. 15 mm to less than 20 mm (45.4% vs. 77.5%, adjusted *p* < 0.001). The optimal threshold for predicting BAD was determined to be 12.1 mm. Patients with infarct sizes of 25 mm or larger had the highest incidence of large-artery atherosclerosis, significantly higher than those with infarct sizes between 10 mm and less than 15 mm (22.4% vs. 7.9%, adjusted *p* < 0.04). The optimal threshold for predicting large-artery atherosclerosis was 14.8 mm. The incidence of cardioembolism did not vary significantly across different infarct sizes.

Table 2 presents the results of the multiple logistic regression analysis, identifying variables significantly associated with END. These include an age over 70 years [adjusted odds ratio (aOR) 2.2; 95% confidence interval (CI) 1.2–4.0], motor tract involvement (aOR 2.3; 95% CI 1.1–4.7), a heart rate above 90 beats per minute (aOR 2.3; 95% CI 1.2–4.3), and a larger infarct size (15 mm to less than 20 mm vs. 10 mm to less than 15 mm; aOR 3.0; 95% CI 1.4–6.3).

Table 3 shows that the multiple logistic regression analysis identified factors significantly associated with a good outcome. These included an age over 70 years (aOR 0.4; 95% CI 0.3–0.5), previous stroke (aOR 0.4; 95% CI 0.3–0.6), atrial fibrillation (aOR 0.6; 95% CI 0.3–1.0), congestive heart failure (aOR 0.2; 95% CI 0.03–0.8), motor tract involvement (aOR 0.6; 95% CI 0.4–0.9), a heart rate above 90 beats per minute (aOR 0.6; 95% CI 0.4–0.9), parent artery stenosis (aOR 0.5; 95% CI 0.3–0.8), and a larger infarct size (10 mm to less than 15 mm vs. less than 10 mm; aOR 0.4; 95% CI 0.3–0.6).

## 4. Discussion

Our study suggests that the sizes of acute SSIs could indicate distinct underlying pathologies. However, the current definition of 20 mm on DWI as the upper limit for small-vessel occlusion is not entirely satisfactory, as it includes a significant number of cases of BAD. A more practical definition of small-vessel disease would be to set the upper limit to 15 mm, but further high-resolution vessel wall imaging is warranted to refine the definition and diagnosis.

Our study demonstrated that an infarct diameter greater than 15 mm was independently linked to a higher incidence of END and a reduced likelihood of favorable functional outcomes. These findings align with previous research indicating that BAD is strongly associated with END, especially in cases of progressive motor deficits, which often result in poor functional recovery [21]. The suggested threshold for the onset of END is an infarct diameter exceeding 15 mm, while the diagnostic threshold for BAD is set to 12 mm. Aligned with a previous review suggesting 15 mm or larger as the definition of BAD, the majority of SSIs measuring 15 mm or more were associated with BAD in our study [4]. A recent study utilizing high-resolution vessel wall imaging for BAD reported a mean infarct diameter of 16.7 ± 4.9 mm in patients with visible atherosclerotic plaques in the parent arteries [22]. These findings underscore the necessity of re-evaluating the diameter criterion for small-vessel occlusion, given that the existing 20 mm threshold falls short. Up to now, various treatment combinations have been explored and suggested to mitigate END in BAD [12]. However, there is currently no therapy with established efficacy for BAD. Therefore, achieving a precise diagnosis of BAD distinct from lacunar infarction is crucial for advancing research and determining the most effective treatment strategies.

BAD is primarily caused by atherosclerosis, particularly when large-caliber penetrating arteries become occluded or narrowed at their origins due to microatheromas or junctional plaques [3]. Several studies have associated intracranial atherosclerotic disease with BAD, identifying it by mild stenosis and a distinct infarct pattern that extends to the basal surface of the parent artery [23]. Even with traditional DWI diagnostic criteria, the detection rate of atherosclerotic plaques along parent artery perforators can reach up to 74% when using high-resolution vessel wall imaging [22]. Importantly, the incidence of parent artery stenosis increases significantly when the arterial diameter exceeds 15 mm. This underscores the value of high-resolution vessel wall imaging in cases where the diameter is greater than 15 mm.

A previous study revealed that perfusion defects in SSIs were associated with BAD, as well as with both EPVS and WMH in the basal ganglia, suggesting a shared underlying pathology [20]. In our study, a larger infarct size was correlated with higher EPVS and WMH scores in the basal ganglia. This may be due to the increased arteriopathy burden in the basal ganglia caused by BAD or intracranial atherosclerosis. This finding aligns with previous research that demonstrated an association between intracranial atherosclerosis and larger WMH volumes [24].

An embolic stroke of undetermined source (ESUS) is typically characterized by a non-lacunar brain infarct without significant proximal arterial stenosis or identifiable cardioembolic sources [25]. According to the TOAST classification, lesions with a diameter of ≥20 mm are currently considered cryptogenic strokes or ESUSs when neither arterial stenosis nor cardioembolic sources are present [5,26]. However, no significant differences in cardioembolism rates were observed across patient groups with different infarct sizes. Prior studies have suggested that only separated subcortical lesions are associated with cardioembolic strokes rather than larger infarct sizes [27]. Thus, using the ESUS classification for lacunar infarctions with a diameter of ≥20 mm may be inappropriate.

The findings of our study suggest that a larger infarction size in acute SSIs is an independent factor that reduces the likelihood of achieving favorable functional outcomes. Additionally, several stroke risk factors—such as previous stroke, atrial fibrillation, and congestive heart failure—also diminished the chances of favorable outcomes, consistent with previous studies [28,29]. These results underscore the importance of the comprehensive evaluation and management of underlying risk factors, even in cases traditionally diagnosed as lacunar infarction. Our study also aligns with earlier research indicating that initial in-hospital heart rate is associated with major adverse cardiovascular events [30].

Although numerous blood biomarkers for stroke have been identified, none have demonstrated adequate sensitivity and specificity for routine clinical use [31,32]. Currently, MRI is the standard tool in clinical practice for identifying acute ischemic stroke rather than blood biomarkers. However, further research is needed to identify reliable serum biomarkers associated with small-vessel disease and to enhance the diagnosis of underlying pathology by combining these biomarkers with MRI.

There are several limitations to the present study. First, we did not utilize high-resolution vessel walls to evaluate the parent arteries of SSIs, which may lead to an inaccurate diagnosis of BAD. While we propose lowering the threshold for small-vessel occlusion from 20 mm to 15 mm, this adjustment is based on current imaging techniques, and future studies should incorporate high-resolution imaging to refine this threshold. Second, our retrospective single-center study, limited to a Taiwanese population, affects the generalizability of our findings. Genetic and environmental factors, as well as local stroke management practices, may not apply universally. A broader multi-center study is needed to validate these results. Moreover, potential confounding factors, such as comorbidities, medication use, and stroke management, may influence the relationship between infarct size and clinical outcomes. Additionally, infarct size can evolve over time, making a single measurement insufficient to fully capture the dynamic changes. Lastly, the absence of significant differences in cardioembolism rates suggests that the ESUS may not be suitable for large lacunar infarctions. However, we did not thoroughly investigate larger infarcts for potential undiagnosed atrial fibrillation using long-term EKG monitoring, which warrants further studies. Despite these limitations, our findings contribute valuable insights to the existing knowledge regarding the association between infarct morphologies and clinical characteristics in acute SSIs.

## 5. Conclusions

Our study suggests that the current definition of a 20 mm axial diameter on DWI as the upper limit for small-vessel occlusion is inadequate, given the observed rates of BAD, END, and functional outcomes. A more practical threshold for small-vessel disease would be an upper limit of 15 mm. However, these findings should be interpreted in the context of the retrospective design and study population. Further multi-center research using high-resolution vessel wall imaging is needed to refine this definition and improve diagnostic accuracy.

## Figures and Tables

**Table 1:** Associations between infarction sizes and clinical characteristics.

Characteristic	Diameter <10 mm	Diameter 10 mm–<15 mm	Diameter 15 mm–<20 mm	Diameter 20 mm–<25 mm	Diameter ≥25 mm	*p*-Value
Number	248	302	182	74	49	
Age	69.5 (60.3–77.8)	69 (61.8–78)	70 (61.8–78)	74.5 (65–79)	66 (58–78)	0.182
Female gender	72 (29.0%)	99 (32.8%)	74 (40.7%)	35 (47.3%)	14 (28.6%)	0.012
Stroke risk factors						
Diabetes mellitus	150 (60.5%)	158 (52.3%)	101 (55.5%)	37 (50.0%)	24 (49.0%)	0.251
Hypertension	206 (83.1%)	245 (81.1%)	147 (80.8%)	62 (83.8%)	41 (83.7%)	0.940
Hypercholesterolemia	82 (33.1%)	125 (41.4%)	92 (50.5%)	33 (44.6%)	26 (53.1%)	0.003
Coronary artery disease	10 (4.0%)	20 (6.6%)	11 (6.0%)	5 (6.8%)	2 (4.1%)	0.697
Atrial fibrillation	26 (10.5%)	21 (7.0%)	16 (8.8%)	4 (5.4%)	5 (10.2%)	0.506
Congestive heart failure	4 (1.6%)	7 (2.3%)	3 (1.6%)	1 (1.4%)	1 (2.0%)	0.965
Old stroke	61 (24.6%)	72 (23.8%)	29 (15.9%)	12 (16.2%)	8 (16.3%)	0.101
TOAST stroke subtypes						<0.001
Large-artery atherosclerosis	30 (12.1%)	24 (7.9%)	27 (14.8%)	12 (16.2%)	11 (22.4%)	
Cardioembolism	27 (10.9%)	23 (7.6%)	13 (7.1%)	5 (6.8%)	4 (8.2%)	
Small-vessel occlusion	186 (75.0%)	255 (84.4%)	137 (75.3%)	0 (0%)	0 (0%)	
Other determined	2 (0.8%)	0 (0%)	0 (0%)	1 (1.4%)	0 (0%)	
Undetermined	3 (1.2%)	0 (0%)	5 (2.7%)	56 (75.7%)	34 (69.4%)	
Stroke information						
Initial NIHSS	3 (1–4)	4 (3–5)	5 (3–6)	5 (3–7)	4 (3–9.5)	<0.001
NIHSS score on 3rd day	2 (1–4)	4 (2.5–6)	5 (3–7)	6 (4–9)	7.5 (4–10)	<0.001
END (∆NIHSS ≥ 4)	16 (6.5%)	22 (7.3%)	30 (16.5%)	16 (21.6%)	13 (26.5%)	<0.001
Axial diameter (mm)	7 (6–8.5)	12 (11–13.1)	17 (16–18.1)	22 (21–23)	29 (26–32)	<0.001
Intravenous alteplase	6 (2.4%)	11 (3.6%)	8 (4.4%)	3 (4.1%)	8 (16.3%)	<0.001
mRS ≥ 1 at 3 months	179 (72.2%)	165 (54.6%)	83 (45.6%)	20 (27.0%)	18 (36.7%)	<0.001
Mortality	2 (0.8%)	2 (0.7%)	0 (0%)	1 (1.4%)	2 (4.1%)	0.551
Neuroimaging information						
Onset–MRI duration (hour)	26.9 (14.8–56.2)	29.2 (18.2–56.2)	33.2 (18.6–57.8)	41.8 (14.9–76.3)	38.3 (25.8–64.1)	0.072
Stroke locations						<0.001
Brainstem	110 (44.4%)	105 (34.8%)	83 (45.6%)	28 (37.8%)	2 (4.1%)	
Thalamus	24 (9.7%)	19 (6.3%)	1 (0.6%)	0 (0%)	0 (0%)	
MCA territory	114 (46.0%)	178 (58.9%)	96 (53.0)	45 (60.8)	47 (95.9)	
Branch atheromatous disease	46 (18.5%)	137 (45.4%)	141 (77.5%)	67 (90.5%)	44 (89.8%)	<0.001
Parent artery stenosis ≥ 50%	38 (15.3%)	32 (10.6%)	32 (17.6%)	14 (18.9%)	11 (22.4%)	0.006
WMH scores of periventricular area	1.8 ± 1.1	2.0 ± 1.1	1.8 ± 1.0	2.3 ± 1.2	2.3 ± 0.9	0.004
WMH scores of basal ganglia	1.6 ± 0.9	1.8 ± 0.9	1.6 ± 0.9	2.2 ± 0.9	2.3 ± 0.8	<0.001
EPVS scores of centrum semiovale	1.8 ± 0.8	1.9 ± 1.0	1.8 ± 0.8	2.2 ± 1.0	1.9 ± 0.8	0.157
EPVS scores of basal ganglia	1.6 ± 0.8	1.8 ± 0.8	1.6 ± 0.8	2.1 ± 0.9	1.6 ± 0.7	0.001
CMBs at lobar areas *	48 (23.4%)	53 (22.9%)	21 (14.6%)	8 (18.2%)	4 (10.8%)	0.120
CMBs at deep areas *	46 (22.4%)	58 (25.1%)	28 (19.4%)	12 (27.3%)	6 (12.2%)	0.549
CMBs at infratentorial areas *	38 (18.5%)	41 (17.7%)	12 (8.3%)	5 (11.4%)	3 (8.1%)	0.034

* CMB data were unavailable in 194 patients. Data were presented as *n* (%), median (interquartile range), or mean ± standard deviation. Abbreviations: CMBs, cerebral microbleeds; END, early neurological deterioration; EPVS, enlarged perivascular space; MCA, middle cerebral artery; MRI, magnetic resonance imaging; mRS, modified Rankin Scale; NIHSS, National Institutes of Health Stroke Scale; WMH, white matter hyperintensity.

**Table 2:** Logistic regression analysis of predictors for early neurological deterioration in patients with small subcortical infarction.

	Simple Logistic Regression			Multiple Logistic Regression		
	Odds Ratio	95% CI	*p*	Adjusted Odds Ratio	95% CI	*p*
Age > 70 years	2.2	1.4–3.4	<0.001	2.2	1.2–4.0	0.008
Female gender	1.4	0.9–2.1	0.133			
Diabetes mellitus	1.3	0.8–1.9	0.311			
Hypertension	1.5	0.8–2.7	0.212			
Hypercholesterolemia	0.8	0.5–1.2	0.221			
Coronary artery disease	1.4	0.6–3.2	0.468			
Previous stroke	1.0	0.6–1.6	0.864			
Atrial fibrillation	1.3	0.6–2.6	0.478			
Congestive heart failure	1.1	0.3–5.0	0.883			
Motor tract involvement	2.2	1.4–3.6	0.001	2.3	1.1–4.7	0.021
Intravenous alteplase	2.0	0.8–4.6	0.124			
Sugar > 180 mg/dL	1.8	1.1–2.9	0.028			
Heart rate > 90/min	2.0	1.2–3.2	0.007	2.3	1.2–4.3	0.009
SBP > 140 mmHg	2.3	1.1–4.9	0.031			
DBP > 90 mmHg	0.9	0.6–1.5	0.776			
Parent artery stenosis	1.5	0.9–2.5	0.146			
Diameter *			<0.001			0.023
<10 mm	1.1	0.6–2.2	0.702	0.7	0.3–1.6	0.335
15 mm–<20 mm	2.9	1.5–5.4	0.001	3.0	1.4–6.3	0.004
20 mm–<25 mm	4.0	1.9–8.5	<0.001	2.8	1.0–7.7	0.045
≥25 mm	5.2	2.3–11.8	<0.001	3.9	1.3–11.6	0.015

* With reference to infarct diameter 10 mm–<15 mm. Abbreviations: SBP, systolic blood pressure; DBP, diastolic blood pressure.

**Table 3:** Logistic regression analysis of predictors for good functional outcomes with mRS ≤ 1 at 3 months in patients with small subcortical infarction.

	Simple Logistic Regression			Multiple Logistic Regression		
	Odds Ratio	95% CI	*p*	Adjusted Odds Ratio	95% CI	*p*
Age > 70	0.4	0.3–0.6	<0.001	0.4	0.3–0.5	<0.001
Female gender	0.7	0.5–1.0	0.022			
Diabetes mellitus	1.0	0.8–1.3	0.958			
Hypertension	0.7	0.5–1.0	0.068			
Hypercholesterolemia	1.2	0.9–1.6	0.152			
Coronary artery disease	0.6	0.4–1.1	0.131			
Previous stroke	0.5	0.3–0.7	<0.001	0.4	0.3–0.6	<0.001
Atrial fibrillation	0.5	0.3–0.8	0.003	0.6	0.3–1.0	0.047
Congestive heart failure	0.1	0.03–0.5	0.005	0.2	0.03–0.8	0.025
Motor tract involvement	0.5	0.4–0.6	<0.001	0.6	0.4–0.9	0.004
Intravenous alteplase	0.8	0.4–1.6	0.590			
Sugar > 180 mg/dL	0.8	0.6–1.2	0.331			
Heart rate > 90/min	0.7	0.5–1.0	0.078	0.6	0.4–0.9	0.012
SBP > 140 mmHg	0.9	0.6–1.2	0.404			
DBP > 90 mmHg	1.2	0.9–1.5	0.311			
Parent artery stenosis	0.5	0.3–0.7	<0.001	0.5	0.3–0.8	0.003
Diameter *			<0.001			<0.001
10 mm–<15 mm	0.5	0.3–0.7	<0.001	0.4	0.3–0.6	<0.001
15 mm–<20 mm	0.3	0.2–0.5	<0.001	0.3	0.2–0.5	<0.001
20 mm–<25 mm	0.1	0.1–0.3	<0.001	0.2	0.1–0.3	<0.001
≥25 mm	0.2	0.1–0.4	<0.001	0.2	0.1–0.3	<0.001

* With reference to infarct diameter < 10 mm. Abbreviations: SBP, systolic blood pressure; DBP, diastolic blood pressure.

## Data Availability

All data inquiries can be directed to the corresponding author.
